# Carotid and femoral Doppler do not allow the assessment of passive leg raising effects

**DOI:** 10.1186/s13613-018-0413-7

**Published:** 2018-05-29

**Authors:** Valentina Girotto, Jean-Louis Teboul, Alexandra Beurton, Laura Galarza, Thierry Guedj, Christian Richard, Xavier Monnet

**Affiliations:** 1 Service de Réanimation Médicale, Hôpital de Bicêtre, Hôpitaux Universitaires Paris-Sud, Insert UMR_999, Université Paris-Sud, Assistance Publique – Hôpitaux de Paris, Le Kremlin-Bicêtre, France; 2 Service de Radiologie, Hôpital de Bicêtre, Hôpitaux Universitaires Paris-Sud, Assistance Publique – Hôpitaux de Paris, Le Kremlin-Bicêtre, France

**Keywords:** Volume expansion, Fluid responsiveness, Hemodynamic monitoring, Cardiac output

## Abstract

**Background:**

The hemodynamic effects of the passive leg raising (PLR) test must be assessed through a direct measurement of cardiac index (CI). We tested whether changes in Doppler common carotid blood flow (CBF) and common femoral artery blood flow (FBF) could detect a positive PLR test (increase in CI ≥ 10%). We also tested whether CBF and FBF changes could track simultaneous changes in CI during PLR and volume expansion. In 51 cases, we measured CI (PiCCO2), CBF and FBF before and during a PLR test (one performed for CBF and another for FBF measurements) and before and after volume expansion, which was performed if PLR was positive.

**Results:**

Due to poor echogenicity or insufficient Doppler signal quality, CBF could be measured in 39 cases and FBF in only 14 cases. A positive PLR response could not be detected by changes in CBF, FBF, carotid nor by femoral peak systolic velocities (areas under the receiver operating characteristic curves: 0.58 ± 0.10, 0.57 ± 0.16, 0.56 ± 0.09 and 0.64 ± 10, respectively, all not different from 0.50). The correlations between simultaneous changes in CI and CBF and in CI and FBF during PLR and volume expansion were not significant (*p* = 0.41 and *p* = 0.27, respectively).

**Conclusion:**

Doppler measurements of CBF and of FBF, as well as measurements of their peak velocities, are not reliable to assess cardiac output and its changes.

**Electronic supplementary material:**

The online version of this article (10.1186/s13613-018-0413-7) contains supplementary material, which is available to authorized users.

## Background

Since it has been demonstrated that fluid overload can be deleterious in patients with acute respiratory distress syndrome [1] and severe sepsis [2], it is of paramount importance to avoid excessive fluid administration in such cases. The decision to give fluids must be guided by a reliable prediction of fluid responsiveness as only 50% of patients respond to fluid administration by increasing cardiac output [3]. In order to predict the response of cardiac output to fluid infusion, the passive leg raising (PLR) test has been validated. It consists in lifting the legs passively at 45° and moving the trunk down horizontally, starting from a semi-recumbent position. By transferring a consistent amount of venous blood from the legs and the splanchnic compartment toward the intrathoracic compartment, it increases the mean systemic pressure [4], the cardiac preload and consequently cardiac output in the case of preload responsiveness of both ventricles [5]. However, it must be coupled with a direct and real-time measurement of cardiac output, which is often invasive [6–8].

The Doppler measurement of blood flow and its velocity in the carotid as well as in the femoral arteries may be interesting for estimating the changes in cardiac output during a PLR test, since changes in arterial blood flow and in cardiac output might be proportional. Nevertheless, contradictory results have been published regarding this issue [9–14].

Our study had two aims. The first was to test whether changes in carotid and femoral Doppler measurements were able to detect a positive PLR test. The second was to investigate the ability of carotid and femoral Doppler measurements to track the changes in cardiac index, during PLR and fluid administration.

## Methods

### Patients

Before starting the study, we obtained the agreement of our institutional review board (*Comité pour la protection des personnes Ile*-*de*-France VI, ref # 2016-A00959-42). All patients or their relatives accepted to participate in the study. It took place at a 25-bed medical intensive care unit of a university hospital between June and November 2016.

Patients were included in the study if they met the following criteria:

- Age ≥ 18 years.
- A PiCCO2 device (Pulsion Medical Systems, Feldkirchen, Germany) already in place for clinical purposes.
- Decision to perform a PLR test made by the attending physicians.

Patients were excluded if the PLR maneuver was contraindicated (intracranial hypertension), if PLR was supposed to be unreliable (venous compression stocking and intraabdominal hypertension) or if it was impossible to perform vascular Doppler measurements.

### Hemodynamic measurements

All patients were equipped with a jugular or subclavian venous catheter and a thermistor-tipped femoral arterial catheter (PV2024, Pulsion Medical Systems). Hemodynamic variables were recorded continuously by using a data acquisition software (HEM 4.2, Notocord, Croissy-sur-Seine, France). Cardiac Index was recorded by the PiCCO Win 4.0 software (Pulsion Medical Systems). For all thermodilution measurements, the results obtained from three consecutive saline boluses were averaged [15, 16].

### Doppler measurements

One investigator (VG) performed all ultrasound measurements. Images were analyzed and measurements were performed offline by two investigators (VG and TG). Ultrasound examination was performed with a CX50 (Philips Healthcare, Eindhoven, The Netherlands) by using a 12–5 MHz flat linear probe.

**Fig. 1:**
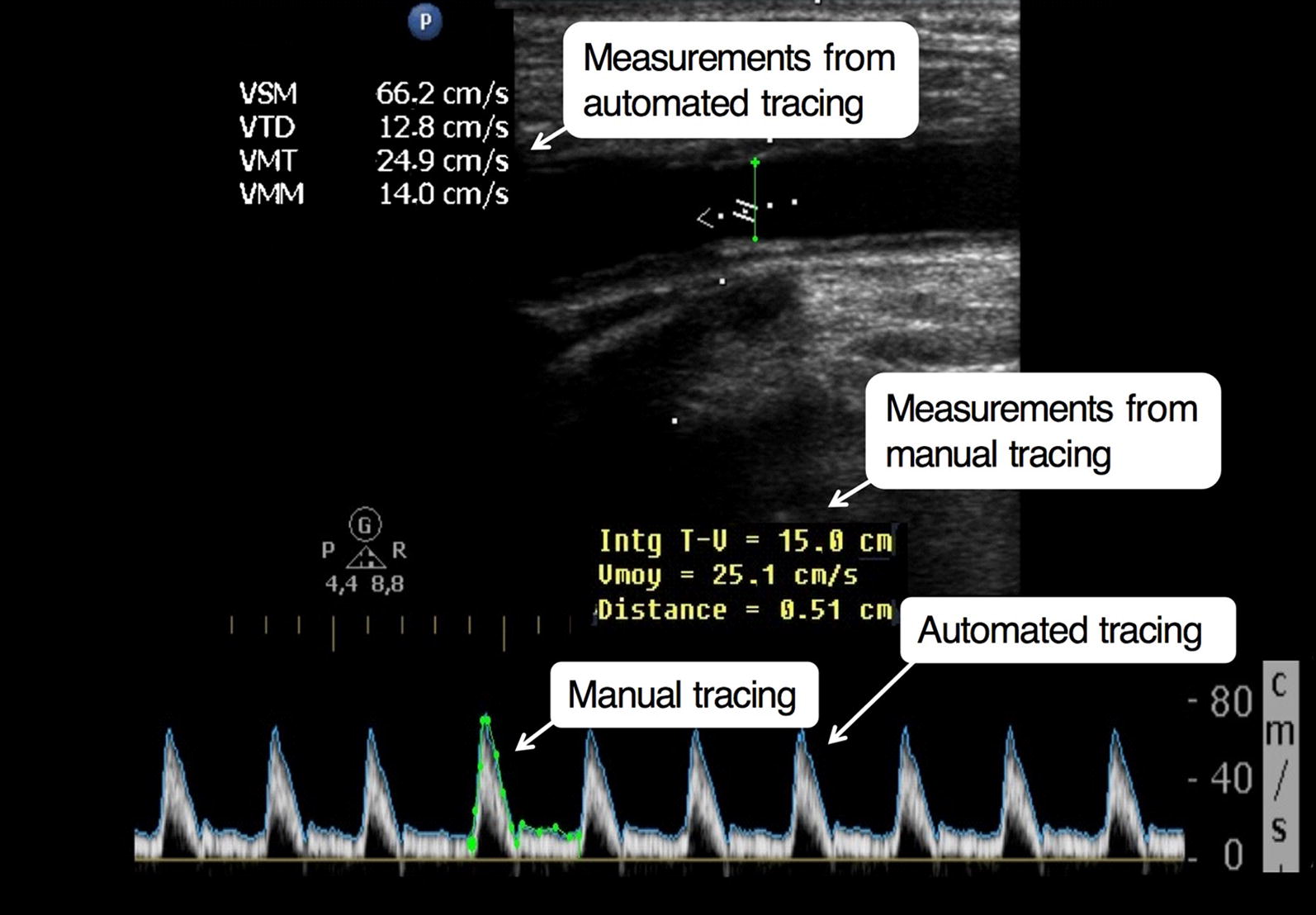
Example of Doppler measurements performed in a patient

At each step of the protocol, we obtained images of the common carotid artery. First, a long-axis view of the carotid artery was obtained approximately 1–2 cm before its bifurcation. We assessed pulsed wave Doppler, the sampling volume being positioned in the middle of the lumen with caliper parallel to blood flow (Fig. 1). Time average mean velocity (TAMEAN) and peak systolic velocity (PSV) were automatically estimated by the echograph software. Velocity-time integral (VTI) was measured by manually tracing the flow envelope for each image (Fig. 1). We kept an insonation angle of 60° between Doppler beam and sample. In longitudinal view, the maximal diameter was measured from intima to intima with an angle of 90° to the vessel.

To determine carotid blood flow, we used two different methods, one based on VTI (cm) and the other on TAMEAN (cm/s):

- Carotid blood flow (mL/min) = TAMEAN × *π r*^2^ × 60.
- Carotid blood flow (mL/min) = VTI × *π r*^2^ × Heart rate (beats/min).

where “*r”* (in cm) represents the radius of the vessel that was assumed to be circular.

In addition, we measured TAMEAN with both narrow and large sampling windows within the arterial lumen, in order to compare two different ways of calculating carotid blood flow.

Measurements were also performed at the level of the common femoral artery before the bifurcation into superficial femoral artery and deep femoral artery. Blood flow, peak systolic velocity and diameter were measured with the same method and formulas as described before. Nevertheless, at this level, the only method that was used to measure femoral blood flow was the one based on VTI. Indeed, the contour of the femoral velocity that was automatically traced by the device for measuring time average mean velocity included both positive and negative values of femoral velocities, eventually providing very low values of TAMEAN. We decided to trace the contour manually, including only the positive values in the measurement of VTI.

### Study design

At baseline, a first set of transpulmonary thermodilution and Doppler measurements were recorded (Additional file 1: Figure S1). Two PLR tests (“PLR1” and “PLR2”) were then consecutively performed because it was not feasible to simultaneously record carotid and femoral Doppler indices during the same PLR test. The PLR position was maintained until the maximal value of pulse contour analysis-derived cardiac index was reached, what always occurs within 1 min [5]. Between the two PLR tests, we waited for approximately 5 min to obtain stable hemodynamic baseline values. Each PLR test was performed as previously described [6]. At its maximum effect, a second set of hemodynamic and Doppler measurements was performed (Additional file 1: Figure S1). The effects of PLR on cardiac output were measured by pulse contour analysis and not by transpulmonary thermodilution because these effects must be assessed by a real-time monitoring technique [6]. In practice, we observed the continuously changing value of pulse contour analysis-derived cardiac index while performing the Doppler measurements. As soon as the cardiac index value started to decrease, we considered that it had reached its maximum. At this precise time, we froze the image of the echograph and performed the Doppler measurements on the values displayed during the previous seconds. If pulse contour analysis-derived cardiac index increased ≥ 10% during the PLR tests, compared to the baseline value, the patient was regarded as responder to the tests [8]. In total, the two PLR tests were performed within 15 min.

After the second PLR, another transpulmonary thermodilution was performed. Then, according to the decision of the clinicians in charge, only responders to the first PLR test were given 500 mL of normal saline over 10 min. All echographic and hemodynamic variables were then recorded at the end of fluid infusion, including transpulmonary thermodilution (Additional file 1: Figure S1). Catecholamines dosages and ventilation settings were kept constant during the study period.

### Data analysis

All data were normally distributed (Kolmogorov–Smirnov test for normality). Date are expressed as mean ± standard deviation (SD) or number and frequency (*n*, %). Comparison between time points of the study was performed using paired Student’s t tests. Comparison between PLR responders and non-responders was performed using two-tailed Student’s *t* tests. Pearson correlation coefficient was calculated to compare carotid/femoral blood flow and cardiac index as well as their relative changes following PLR and fluid infusion. A receiving operating characteristics (ROC) curve was constructed to evaluate the ability of the PLR-induced changes in carotid and femoral blood flows and velocities to detect responsiveness to PLR. The inter- and intraindividual variability of carotid Doppler measurements were also calculated. Considering a *α*-risk of 20% and a *β*-risk of 10%, to evidence an increase in 20% of carotid and femoral blood flows during PLR [9, 10], we planned to include 50 cases in the study. Statistical significance was defined by a *p* value < 0.05. The statistical analysis was performed using MedCalc 11.6.0 software (MedCalc, Mariakerke, Belgium).

## Results

### Patient characteristics

**Fig. 2:**
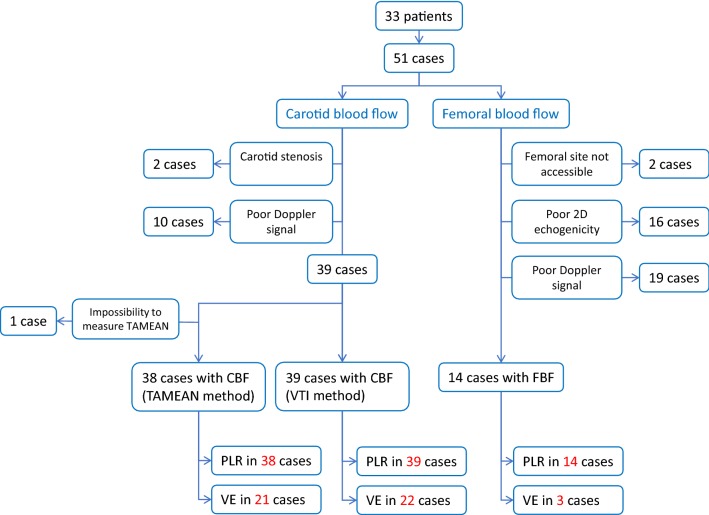
Flowchart. *CBF* carotid blood flow, *PLR* passive leg raising, *TAMEAN* time average mean velocity, *VE* volume expansion, *VTI* velocity time integral

Thirty-three patients were included in the study. Patients could be included more than once at different days, so that we collected 51 cases in total, which were considered as individual cases (Fig. 2). Their characteristics are summarized in Table 1.

**Table 1:** Baseline patient characteristics

Gender (male)	22 (67%)
Age (years)	67 ± 14
Weight (kg)	68 ± 12
Height (cm)	165 ± 9
SAPS II	62 ± 19
Diagnostic	
Septic shock	16 (49%)
Cardiogenic shock	7 (21%)
ARDS	6 (18%)
Coma	2 (6%)
Pancreatitis	1 (3%)
Acute renal failure	1 (3%)
LVEF < 50%	8 (24%)

*N* = 33

Data are presented as mean ± standard deviation or number (percentage)

*SAPS II* simplified acute physiology score, *ARDS* acute respiratory distress syndrome, *LVEF* left ventricular ejection fraction

At the time of inclusion, in 48 (94%) cases, patients were intubated and ventilated in the volume-controlled mode. Patients received catecholamines in 46 (90%) cases (norepinephrine alone in 41 cases, dobutamine and norepinephrine in three cases, dobutamine alone in two cases).

### Feasibility of carotid and femoral Doppler examination

Among all carotid Doppler measurements, two cases were excluded because of carotid stenosis and 10 because of poor image quality that prevented to reliably trace the contour of the signal (Fig. 2). Among the remaining 39 cases, in one case we could not assess carotid blood flow by TAMEAN (Fig. 2).

Among all cases, two were excluded because the femoral site was not accessible for performing Doppler measurement (obesity), 16 cases were excluded because of a poor 2D echogenicity that prevented to precisely define the intima edge of the femoral artery and 19 cases because of poor quality of the Doppler signal (Fig. 2).

An increase in cardiac index ≥ 10% during the first PLR predicted fluid responsiveness with a positive predictive value of 93%. The specificity, sensitivity and negative predictive of PLR as a predictor of the response to fluid infusion value could not be calculated since we performed fluid infusion only in patients with a positive PLR test. An increase in cardiac index ≥ 10% during the second PLR predicted fluid responsiveness with the same positive predictive value because both PLR tests exerted similar effects on cardiac index.

**Fig. 3:**
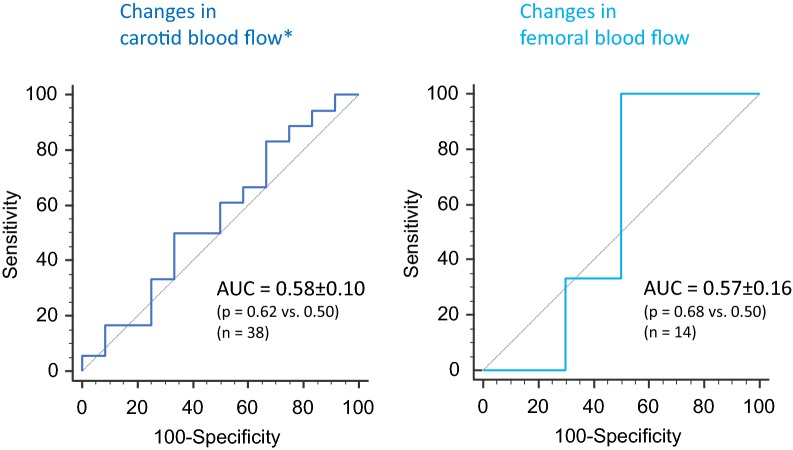
Receiver operating characteristic curves describing the ability of changes in carotid femoral blood flows to detect a positive response of cardiac index to a passive leg raising test (increase ≥ 10%). *AUC* area under the curve. Asterisks results are provided for carotid blood flow measured by the velocity time integral method

The results of ROC curves analysis are presented in Additional file 1: Table S1 and Fig. 3. Neither the changes in carotid blood flow measured with the VTI method nor the carotid blood flow measured the TAMEAN method or the carotid PSV could detect a positive response to the PLR1 test. Neither the changes in femoral blood flow measured with the VTI method nor the femoral PSV could detect a positive response to the PLR2 test (Additional file 1: Table S1, Fig. 3). Results were not different when the analysis was performed with only the first case measured in each of the patients who had been included several times in the study (data not shown).

### Relationship between cardiac index and carotid Doppler measurements in absolute values and relative changes

Absolute values of carotid blood flow and of PSV as well as the ratio of carotid blood flow over cardiac index during each study step are reported in Table 2.

**Table 2:** Hemodynamic and Doppler measurements

	Baseline 1	PLR1	Baseline 2	PLR2	Baseline 3	After fluid infusion
Heart rate (beats/min)						
PLR responders (*n* = 27)	91 ± 19	92 ± 22	89 ± 17	93 ± 17	92 ± 15	93 ± 15
PLR non-responders (*n* = 24)	91 ± 18	91 ± 17	87 ± 14	92 ± 14	89 ± 19	–
Systolic arterial pressure (mmHg)						
PLR responders (*n* = 27)	117 ± 26	129 ± 32*	115 ± 25	130 ± 34^#^	115 ± 32	129 ± 33^$^
PLR non-responders (*n* = 24)	125 ± 21	130 ± 24*	122 ± 18	127 ± 20^#^	125 ± 20	–
Diastolic arterial pressure (mmHg)						
PLR responders (*n* = 27)	57 ± 13	62 ± 11*	52 ± 16	62 ± 11^#^	57 ± 14	63 ± 18^$^
PLR non-responders (*n* = 24)	61 ± 9	64 ± 11*	60 ± 7	64 ± 9^#^	61 ± 10	–
Central venous pressure (mmHg)						
PLR responders (*n* = 27)	11 ± 4	14 ± 5*	9 ± 4	15 ± 5^#^	11 ± 4	12 ± 4^$^
PLR non-responders (*n* = 24)	10 ± 6	14 ± 6*	10 ± 6	13 ± 7^#^	10 ± 6	–
Cardiac index (L/min/m^2^)						
PLR responders (*n* = 27)	3.11 ± 1.21	3.62 ± 1.29*	2.98 ± 1.15	3.63 ± 1.27^#^	2.91 ± 0.91	3.53 ± 1.16^$^
PLR non-responders (*n* = 24)	3.16 ± 1.07	3.23 ± 1.12	3.14 ± 1.10	3.23 ± 1.24^#^	3.17 ± 1.13	–
Carotid artery flow (TAMEAN) (mL/min)						
PLR responders (*n* = 21)	371 ± 138	407 ± 144	–	–	335 ± 118	390 ± 141^$^
PLR non-responders (*n* = 17)	293 ± 128	344 ± 159	–	–	321 ± 130	–
Carotid artery flow (VTI) (mL/min)						
PLR responders (*n* = 21)	615 ± 194	674 ± 202	–	–	601 ± 214	690 ± 221^$^
PLR non-responders (*n* = 17)	593 ± 225	617 ± 218	–	–	577 ± 227	–
Carotid PSV (cm/s)						
PLR responders (*n* = 22)	88 ± 23	82 ± 21	–	–	81 ± 22	88 ± 22
PLR non-responders (*n* = 17)	83 ± 30	77 ± 28	–	–	82 ± 23	–
Cardiac index to common carotid artery (TAMEAN) (%)						
PLR responders (*n* = 21)	13 ± 5	12 ± 4	–	–	12 ± 3	13 ± 5
PLR non-responders (*n* = 17)	9 ± 2	10 ± 3	–	–	10 ± 3	–
Femoral artery flow (VTI) (mL/min)						
PLR responders (*n* = 3)	–	–	408 ± 331	404 ± 319	433 ± 400	733 ± 800
PLR non-responders (*n* = 11)	–	–	368 ± 126	386 ± 127	382 ± 78	–
PSV femoral (cm/s)						
PLR responders (*n* = 17)	–	–	84 ± 28	111 ± 45^#^	77 ± 28	86 ± 31^$^
PLR non-responders (*n* = 18)	–	–	78 ± 17	89 ± 17	78 ± 20	–

Data are presented as mean ± standard deviation. PLR responders: cases with increase in pulse contour analysis-derived cardiac index ≥ 10% during passive leg raising, PLR non-responders: cases with increase in pulse contour analysis-derived cardiac index < 10% during passive leg raising

*TAMEAN* time average mean velocity, *PSV* peak systolic velocity

* *p* < 0.05 versus Baseline 1; ^#^ *p* < 0.05 versus Baseline 2; ^$^ *p* < 0.05 versus Baseline 3

For TAMEAN, the inter-individual variability was 8.9 ± 8.7% and the intraindividual variability was 12.7 ± 12.2%. For PSV, the inter-individual variability was 5.0 ± 4.1% and the intraindividual variability was 2.2 ± 1.7%. No difference was found between values of carotid blood flow calculated from TAMEAN sampled in large and narrow sampling windows (*p* = 0.28).

Considering all measurements at different study steps (Fig. 2), only weak correlations were found between absolute values of cardiac index and absolutes values of carotid blood flow calculated from TAMEAN (*n* = 135; *r* = 0.54, *p* < 0.01) (Additional file 1: Figure S2) and absolutes values of carotid PSV (*n* = 139; *r* = 0.26, *p* < 0.01). Absolute values of carotid blood flow calculated with TAMEAN were almost systematically lower than the corresponding values calculated with VTI (data not shown).

Considering all changes observed during the first PLR test (*n* = 38) and fluid infusion (*n* = 21) (Fig. 2), we found no correlation between changes in cardiac index and changes in carotid blood flow calculated from TAMEAN (*n* = 59; *r* = 0.07, *p* = 0.61) and between changes in cardiac index and changes in carotid blood flow calculated from VTI (*n* = 61; *r* = 0.11, *p* = 0.41). The ability of changes in carotid blood flow calculated from VTI and TAMEAN to detect changes in cardiac index are illustrated by 4-box tables in Additional file 1: Table S2. Results were not different when the analysis was performed with only the first case measured in each of the patients who had been included several times in the study (data not shown).

### Relationship between cardiac index and femoral Doppler measurements in absolute values and relative changes

Considering all measurements at different study steps (*n* = 45, Fig. 2), a weak correlation was found between absolute values of femoral blood flow and cardiac index (*r* = 0.21, *p* = 0.17). Still considering all measurements performed at the femoral level at different study steps (*n* = 118, Fig. 2), a weak correlation was found between absolute values of PSV and cardiac index (*r* = 0.32, *p* < 0.01) (Additional file 1: Figure S3).

Considering all changes observed during the second PLR test and during fluid infusion (*n* = 17, Fig. 2), the correlation coefficient between changes in femoral blood flow and changes in cardiac index was *r* = 0.28 (*p* = 0.27). The ability of changes in carotid blood flow calculated from VTI and TAMEAN to detect changes in cardiac index are illustrated by 4-box tables in Additional file 1: Table S2. Results were not different when the analysis was performed with only the first case measured in each of the patients who had been included several times in the study (data not shown).

## Discussion

The main finding of our study is that carotid and femoral blood flow and their peak velocities did not allow the detection of a positive PLR test and that their changes were not correlated with the simultaneous changes in cardiac index.

The previous results regarding the ability of Doppler measurements of peripheral arteries to estimate cardiac output and its changes are very controversial. Marik et al. [9] have demonstrated an excellent ability of changes in carotid blood flow to detect the PLR effects. Nevertheless, the authors used bioreactance as the reference for measuring cardiac output, while the accuracy of this technique has been seriously questioned [17, 18]. In a study by Préau et al. [10], the variation in femoral artery peak systolic velocity during PLR could reliably predict fluid responsiveness in critically ill patients. Nevertheless, in this study, the carotid blood flow was not investigated and, on the femoral site, only the peak systolic velocity was investigated [10]. Moreover, in this study, the diagnostic threshold that they measured for PLR-induced increases in femoral peak velocity was 8%, while the inter-observer variability of this variable was as large as 8.4 ± 9.2%.

In contrast with these results, other studies in cardiac surgery patients [11, 12] and healthy volunteers [13, 14] showed that the correlation between changes in cardiac output and in common carotid blood flow either was weak or had wide limits of agreement. Our results corroborate these negative studies. Rohering et al. [12] found a strong correlation between absolute values and changes of carotid blood flow and cardiac index. However, limits of agreement in the Bland–Altman analysis (± 20%) were so wide that they concluded that carotid Doppler should not replace direct cardiac output monitoring, especially for performing the PLR test [12]. In the study by Peatchy et al. [13], changes in carotid diameter were not measured during PLR. We measured this diameter in our study, but this did not improve the reliability of the estimation of cardiac index by carotid blood flow.

Several reasons may explain these findings. First, regarding the carotid Doppler signal, from a physiological point of view, the proportion of cardiac output that is directed toward the carotid artery may vary depending on cerebral blood flow regulation, impairing the correlation between carotid blood flow and cardiac output and controversial results have been reported regarding this point [19–24]. Second, another explanation may be the lack of reliability of the carotid and femoral Doppler measurements themselves. In the literature, we could not find a gold standard to calculate femoral and carotid blood flows. Many different methods exist [25], and they provide discordant results [26] with numerous sources of error [27]. In our study, absolute values of carotid blood flow measured by TAMEAN were in accordance with values shown in literature [22], but they were almost systematically half of the values obtained from VTI. Even in patients that had not been excluded from the study, the echogenicity and the quality of the Doppler signal prevented to obtain precise measurements in many cases, especially at the femoral level. This likely led to errors in the measurement of the vessel diameter and hence to even larger miscalculations of blood flow values, as the squared value of arterial diameter is taken into account for measuring them. The measurement of femoral blood flow was impeded by the fact that, at this level, the anatomical landmarks tended to change with PLR. This likely explained the large intra- and inter-variability, indicating that these techniques are not suitable for the precise measurement of changes of small amplitude. Finally, access to the femoral site was difficult in obese patients, such that two of such patients were excluded. Eventually, we obtained a limited number of Doppler measurements for femoral artery. This fact may be enough to conclude that the method is not adapted to current practice in the ICU setting.

## Limitations

First of all, we obtained only a limited number of measurements of Doppler variables, what has reduced the power of our analysis. Nevertheless, given the poor results we observed, it is unlikely that including more patients would have led to better results. Regarding femoral measurements, the fact that it was impossible to acquire them in a so large proportion of patients itself indicates that the technique is not appropriate. Second, some patients have been included several times in the study. Nevertheless, the analysis performed with only the first measurement performed in these patients did not show different results from the main analysis. Third, Doppler measurements were performed on one side only, while the opposite one may have provided better results. Fourth, although we took the precaution to exclude it, it is still possible that a mild degree of arterial stenosis may have influenced the relationship between cardiac output and arterial flow. Fifth, Doppler examinations were performed at the bedside in the ICU, while measurements performed in an echographic laboratory could provide more reliable measurements. Nevertheless, our methodology reflects the real-life practice. Finally, fluid infusion was not performed in non-responders, so that we could not assess the specificity and sensitivity of PLR-induced changes in arterial blood flows or velocity to assess fluid responsiveness. Nevertheless, given the poor reliability of Doppler measurements obtained in PLR responders, it is very likely that they did not perform better in PLR non-responders.

## Conclusions

Carotid and femoral blood flows and peak systolic velocities were not reliable to assess the effects of a PLR test. These methods were not reliable to estimate cardiac output and its variations in intensive care patients. Many technical and physiological reasons may explain this lack of reliability.

## Additional file

**Additional file 1: Table S1.** Ability of different Doppler variable to detect a positive passive leg raising test. **Table S2.** Diagnostic ability of changes in carotid and femoral blood flows to detect changes in cardiacindex ≥ 10% and ≥ 15%. **Figure S1.** Study design. **Figure S2.** Correlation between absolute values of carotid blood flow (measured by TAMEAN) and of cardiac index, *n* = 135 (*n* = 38 before PLR, 38 during passive leg raising (PLR), 38 after PLR and 21 after volume expansion = 135 in total). **Figure S3.** Correlation between absolute values of femoral blood flow and of cardiac index, *n* = 45 ( *n* = 14 before PLR, 14 during passive leg raising (PLR), 14 after PLR and 3 after volume expansion = 45 in total).

## Abbreviations

AUROC: area under the receiver operating characteristics curve
PLR: passive leg raising
PSV: peak systolic velocity
ROC: receiving operating characteristics
SD: standard deviation
TAMEAN: time average mean velocity
VTI: velocity–time integral
