# Supplementary material for: Carotid and femoral Doppler do not allow the assessment of passive leg raising effects
Source: Ann Intensive Care. 2018 May 29;8:67. doi: 10.1186/s13613-018-0413-7 (PMC5975047; doi:10.1186/s13613-018-0413-7)
Supplement: Supplementary file 1 — Additional file 1: Table S1. Ability of different Doppler variable to detect a positive passive leg raising test. Table S2. Diagnostic ability of changes in carotid and femoral blood flows to detect changes in cardiacindex ≥ 10% and ≥ 15%. Figure S1. Study design. Figure S2. Correlation between absolute values of carotid blood flow (measured by TAMEAN) and of cardiac index, n = 135 (n = 38 before PLR, 38 during passive leg raising (PLR), 38 after PLR and 21 after volume expansion = 135 in total). Figure S3. Correlation between absolute values of femoral blood flow and of cardiac index, n = 45 ( n = 14 before PLR, 14 during passive leg raising (PLR), 14 after PLR and 3 after volume expansion = 45 in total). [file 13613_2018_413_MOESM1_ESM.docx]

Carotid and femoral Doppler do not allow the assessment of the effects of passive leg raising

by

Valentina GIROTTO^1^, MD; Jean-Louis TEBOUL^1^, MD, PhD; Alexandra BEURTON^1^, MD; Laura GALARZA^1^, MD; Thierry GUEDJ^2^, MD; Christian RICHARD^1^, MD ; Xavier MONNET^1^, MD, PhD.

1. Service de réanimation médicale, Hôpital de Bicêtre, Hôpitaux universitaires Paris-Sud, Assistance publique – Hôpitaux de Paris, Le Kremlin-Bicêtre, France

Inserm UMR S_999, Université Paris-Sud, Le Kremlin-Bicêtre, France

2. Service de radiologie, Hôpital de Bicêtre, Hôpitaux universitaires Paris-Sud, Assistance publique – Hôpitaux de Paris, Le Kremlin-Bicêtre, France

Supplemental material

| Tables  **** |  |  |  |  |  |  |
| --- | --- | --- | --- | --- | --- | --- |

Figures

**Figure S1**

Study design

CI: cardiac index, PLR: passive leg raising, TPTD: transpulmonary thermodilution

**Figure S2**

Correlation between absolute values of carotid blood flow (measured by TAMEAN) and of cardiac index, n=135 (n=38 before PLR, 38 during passive leg raising (PLR), 38 after PLR and 21 after volume expansion = 135 in total).

**Figure S3**

Correlation between absolute values of femoral blood flow and of cardiac index, n=45 (n=14 before PLR, 14 during passive leg raising (PLR), 14 after PLR and 3 after volume expansion = 45 in total)
